# Impact of Host Immune Status on Discordant Anti-SARS-CoV-2 Circulating B Cell Frequencies and Antibody Levels

**DOI:** 10.3390/ijms222011095

**Published:** 2021-10-14

**Authors:** Frédéric Coutant, Jean-Jacques Pin, Florence Morfin-Sherpa, Tristan Ferry, Stéphane Paul, Bruno Pozzetto, Myriam Normand, Pierre Miossec

**Affiliations:** 1 Immunogenomics and Inflammation Research Team, University of Lyon, Edouard Herriot Hospital, 69003 Lyon, France; frederic.coutant@univ-lyon1.fr; 2 Immunology Department, Lyon-Sud Hospital, Hospices Civils of Lyon, 69310 Pierre-Bénite, France; 3 Dendritics-Edouard Herriot Hospital, 69003 Lyon, France; jean_jacques_pin@hotmail.fr; 4 Virology Department, Infective Agents Institute, National Reference Center for Respiratory Viruses, North Hospital Network, 69004 Lyon, France; florence.morfin-sherpa@chu-lyon.fr; 5 Virpath Team, CIRI, INSERM U1111, CNRS UMR5308, ENS Lyon, University Claude Bernard Lyon 1, 69100 Lyon, France; 6 Department of Infectious and Tropical Diseases, Hospices Civils of Lyon-Croix-Rousse Hospital, 69004 Lyon, France; tristan.ferry@chu-lyon.fr; 7 Stapath Team, CIRI, INSERM U1111, CNRS, UMR5308, ENS Lyon, University Claude Bernard Lyon 1, 69100 Lyon, France; 8 Department of Immunology, University Hospital of Saint-Etienne, 42055 Saint-Etienne, France; stephane.paul@chu-st-etienne.fr; 9 GIMAP Team, CIRI, INSERM U1111, CNRS, UMR530, University Claude Bernard Lyon 1, CIC 1408 Vaccinology, 42023 Saint-Etienne, France; bruno.pozzetto@univ-st-etienne.fr; 10 Department of Infectious Agents and Hygiene, University Hospital of Saint-Etienne, 42055 Saint-Etienne, France; 11 SAINBIOSE, INSERM, U1059, University of Lyon, 42270 Saint-Etienne, France; myriam.normand@univ-st-etienne.fr; 12 Department of Immunology and Rheumatology, Edouard Herriot Hospital, 69003 Lyon, France

**Keywords:** SARS-CoV-2, COVID-19, anti-SARS-CoV-2, B cell repertoire, human monoclonal antibody

## Abstract

Individuals with pre-existing chronic systemic low-grade inflammation are prone to develop severe COVID-19 and stronger anti-SARS-CoV-2 antibody responses. Whether this phenomenon reflects a differential expansion of antiviral B cells or a failure to regulate antibody synthesis remains unknown. Here, we compared the antiviral B cell repertoire of convalescent healthcare personnel to that of hospitalized patients with pre-existing comorbidities. Out of 277,500 immortalized B cell clones, antiviral B cell frequencies were determined by indirect immunofluorescence screening on SARS-CoV-2 infected cells. Surprisingly, frequencies of SARS-CoV-2 specific clones from the two groups were not statistically different, despite higher antibody levels in hospitalized patients. Moreover, functional analyses revealed that several B cell clones from healthcare personnel with low antibody levels had neutralizing properties. This study reveals for the first time a key qualitative defect of antibody synthesis in severe patients and calls for caution regarding estimated protective immunity based only on circulating antiviral antibodies.

## 1. Introduction

Severe acute respiratory syndrome coronavirus-2 (SARS-CoV-2) infection is characterized by a large heterogeneity in disease severity, ranging from asymptomatic to fatal cases, and in antiviral immune responses [1]. Several quantitative and qualitative defects affect peripheral T- and B cells in the most severe forms of coronavirus disease 2019 (COVID-19) [2,3,4]. In these patients, the abnormal stability of CD8+ and CD4+ T cell activation suggests a functional T cell dysregulation and disruption of a coordinated T cell–B cell interaction, resulting in higher humoral immune responses [5,6,7,8]. This unbalanced crosstalk takes place particularly in infected individuals with comorbidities, such as obesity, and/or older people [9,10,11,12,13]. The loss of functional T cell/B cell interaction is notably shown by higher levels of delayed anti-SARS-CoV-2 antibodies (Ab). It leads to the emergence of oligoclonal B cell populations with high serum oligoclonality, and to the presence of autoAb, particularly against coagulation and vessel targets [14,15]. These comorbidities have in common chronic systemic low-grade inflammation, with an abnormal production of pro-inflammatory cytokines and the impairment of T cell mediated immune response involved in host defense [16,17,18,19]. These immune defects affect several critical pathways that support the cooperation between T cells and B cells, such as the CD40/CD40 ligand pathway, which are probably even more deregulated by the inflammation caused by the SARS-CoV-2 infection.

A still pending question is whether the high levels of anti-SARS-CoV-2 Ab observed in patients after severe COVID-19 reflect a differential expansion of antiviral B cells or a failure to regulate appropriately Ab synthesis in individuals with pre-existing immune dysfunctions. The analysis of the functional B cell repertoire of patients who have recovered is a critical step for providing answers to this question. To this end, a classical approach would consist in isolating antigen-specific B cells, and the cloning of their heavy and light chains to produce a synthetic monoclonal Ab in a heterologous system. This method is efficient for the exploration of the B cell repertoire at a single cell level, but as antigen-specific B cells are isolated, only the cells that are reactive to the viral antigen are selected, and subsequently analyzed. Moreover, it does not enable the generation of genuine antiviral Ab naturally produced by B cells, which can bias the functional interpretation of the B cell repertoire.

A more extensive and exhaustive approach was applied here to compare the B cell repertoire of convalescent patients recovering of a severe form of the disease (SP) to the repertoire of convalescent healthcare personnel (HP) without pre-existing immune system alterations, who recovered from a mild form of COVID-19. This approach relies on the activation of the entire B cell repertoire present in the blood sample via the CD40 system, which results in a transient cell proliferation and secretion of immunoglobulins [20]. This transient state is then maintained by the immortalization of the cells with the Epstein–Barr virus (EBV), generating stable cell lines secreting Ab. By this way, the immortalization yields are 10,000 to 100,000 times higher than the yields obtained with a classical transformation by EBV, allowing the generation of a large spectrum of B cell clones, representative of the global B cell repertoire [21]. Among all of these clones, virus-specific clones were identified by screening the supernatants on SARS-CoV-2 infected cells by indirect immunofluorescence in order to avoid the restriction bias inherent to a screening process based on a recombinant viral antigen. Several cycles of subcloning are then necessary to obtain a monoclonal stage, which is validated by isotyping and sequencing.

By this approach, more than 277,000 clone supernatants were screened on SARS-CoV-2 infected cells by indirect immunofluorescence. This strategy allowed for the first time to establish the unbiased frequencies of SARS-CoV-2–specific B cells of convalescent SP and HP, which were then compared to plasma levels of antiviral Ab.

## 2. Results

### 2.1. Plasma Titers of Anti-SARS-CoV-2 Antibodies

As extensive functional analysis of the B cell repertoire is a very laborious approach, the present study was performed on a limited number of representative individuals. From 30 April 2020 to 18 May 2020, five convalescent SP that required hospitalization and five convalescent HP who recovered from a mild form of COVID-19 were enrolled in this study. All were recruited from the same hospital unit and had reverse transcription PCR confirmed SARS-CoV-2 infection. Infections by SARS-CoV-2 of HP were most likely acquired during routine care of SP, providing the opportunity to restrain differences between the two groups, due to virus-specific factors.

There was no significant difference between the two groups with respect to sex (*p* = 0.524, Table 1 and Supplementary Table S1).

**Figure 1:**
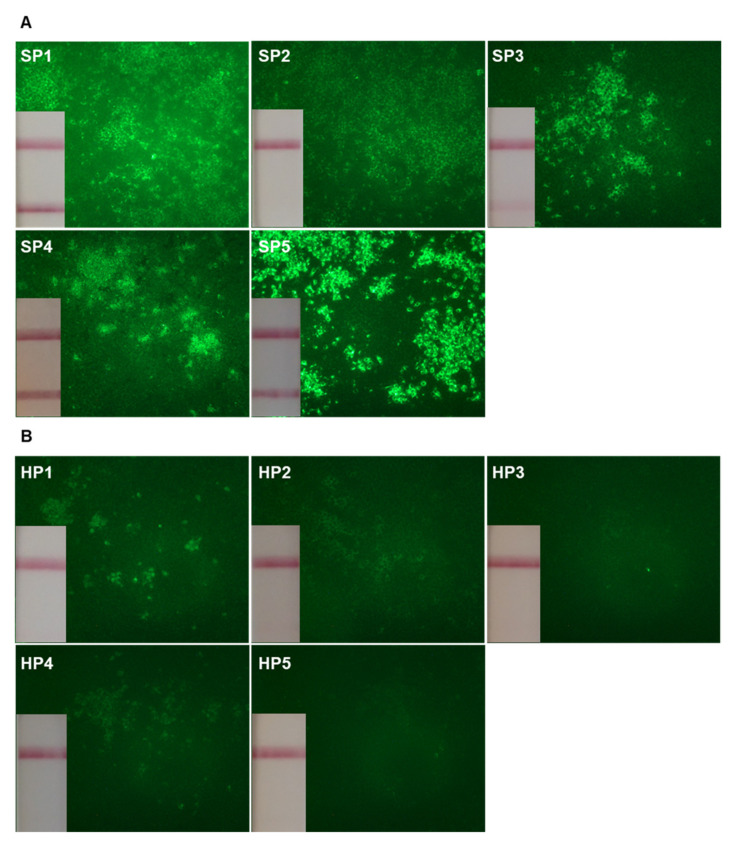
Detection of antiviral antibodies in the plasma of convalescent COVID-19 patients. Indirect immunofluorescence staining was performed on fixed SARS-CoV-2 infected BGM cells, using diluted plasma from severe patients (**A**) and from convalescent healthcare personnel (**B**) of the disease. For each plasma tested, the results of the Wantai SARS-CoV-2 Ab rapid test obtained are inserted at the bottom left of the immunofluorescence image.

Advanced age and hypertension were the two risk factors for severe disease preferentially found in the SP group, with 4/5 over 65 years old in the group SP versus 0/5 in the group HP (*p* = 0.048), and 5/5 hypertensive SP versus 1/5 hypertensive HP (*p* = 0.048). Although statistically non-significant, the number of diabetic and obese patients were higher in the SP than in the HP group (2/5 versus 0/5, *p* = 0.444 and 3/5 versus 1/5, *p* = 0.524 respectively). HP samples were collected later after the onset of symptoms, compared to SP samples (39 (27–45) vs. 20 (15–38) days, *p* = 0.032, Table 1). In order to determine the titer of all circulating antiviral Ab, plasma samples were evaluated by indirect immunofluorescence for SARS-CoV-2 reactivity on fixed BGM cells infected with the virus (wild-type Wuhan SARS-CoV-2). By this method, we found higher titers of plasma antiviral Ab in SP, compared to HP (2.3 (2.2–2.6) and 1.7 (1.4–2.2) respectively, *p* = 0.024, Table 1, Figure 1A,B).

The presence of specific plasma Ab against the spike protein receptor binding domain (RBD) and against the nucleocapsid proteins (NP) were then evaluated with three platforms widely used in diagnostic laboratories (the Wantai SARS-CoV-2 rapid test for the detection of anti-RBD total Ab, and two Wantai ELISA for the detection of anti-RBD and anti-NP total Ab). Importantly, samples from the HP group were negative when tested with the Wantai SARS-CoV-2 rapid test, as well as one plasma from the SP group, confirming that pauci-symptomatic individuals have lower, or even undetectable, levels of specific Ab (Figure 1B). Consistent with other reports [23] and as shown in Table 1, all measurements showed higher levels of antiviral Ab in SP, compared to HP (rapid test anti-RBD Ab: 3 (0–4) vs. 0 (0–1) *p* = 0.048, ELISA anti-RBD Ab: 1.9 (1.7–2.2) vs. 0.9 (0.8–0.9) *p* = 0.008, ELISA anti-NP Ab: 2.8 (2.6–3.6) vs. 1.7 (0.1–2.4) *p* = 0.008).

### 2.2. Frequencies of Circulating SARS-CoV-2 Reactive B cells

A pending question is whether the high levels of anti-SARS-CoV-2 Ab observed in SP compared to HP reflect a differential expansion of antiviral B cells or a failure to regulate appropriately Ab synthesis. To address this question, a library of immortalized circulating B cells reflecting the entire circulating B cell repertoire of each patient was obtained, using an optimized technology based on EBV transformation and CD40/CD40 ligand stimulation. B cell clone supernatants were then screened by indirect immunofluorescence for the presence of Ab reacting on fixed BGM cells, infected with the wild-type SARS-CoV-2. The revelation step was performed with FITC anti-human IgG, A, M Ab, to identify all the antiviral B cells present in the initial repertoire. In total, 277,500 B cell clones were analyzed (133,000 derived from SP samples and 144,500 from HP samples). Surprisingly, the frequencies of antiviral B cell clones were not statistically different between the two groups (0.5 (0.3–1.8) vs. 1.0 (0.3–9.0) for HP and SP, respectively, *p* = 0.309, Table 1). Thus, the frequency of circulating SARS-CoV-2 reactive B cells does not correlate with the plasma levels of antiviral Ab (r^2^ = 0.0002799, *p* = 0.96, Figure S1).

### 2.3. Neutralization Activity of Circulating SARS-CoV-2 Specific B Cell Clones

The absence of quantitative difference in frequency of circulating antiviral B clones regardless of the severity of the disease prompted us to evaluate the neutralizing activity of some of these clones. After successive steps of cloning and enrichment, a total of 204 culture supernatants of SARS-CoV-2 reactive B cell clones were tested for their abilities to inhibit the infection of BGM cells by live SARS-CoV-2. Among the 204 supernatants tested, four B cell clones from HP, and seven clones from SP inhibited SARS-CoV-2 infection of BGM cells (Table 2).

This result was unexpected, especially for the convalescent HP group, since it contrasts with the plasma levels of anti-RBD Ab of HP, which were very low or even undetectable, depending on the serological test used.

The analysis of the isotype of the 11 neutralizing B cell clones did not reveal any dominant immunoglobulin class. The antigenic specificity of the Ab was determined by evaluating their reactivities against the following antigens from the wild-type SARS-CoV-2: RBD, S1, S2, NP, envelope, helicase, and papain-like protease protein. Among the 11 clones, 6 were specific for the RBD domain, 1 was specific of the S1 domain of the spike protein but did not recognize the RBD domain, and 4 clones stained SARS-CoV-2 infected BGM cells, but were not reactive against the tested antigens.

## 3. Discussion

These results demonstrate that the frequency of circulating SARS-CoV-2 reactive B cells does not correlate with the plasma levels of antiviral Ab in both SP and HP. This result was rather unexpected since one would expect that the higher titers of antiviral Ab in SP simply reflected higher frequencies of SARS-CoV-2 reactive B cells, compared to convalescent HP. To date, and to our knowledge, this study is the first one to evaluate the entire SARS-CoV-2–specific B cell repertoire of convalescent SP and HP. Indeed, the frequencies of SARS-CoV-2 specific B cells defined so far in convalescent patients are in fact restricted to the frequencies of B cells specific to spike, RBD or nucleocapsid [24,25]. The frequencies determined by these approaches range from 0.005% to 0.1%, and are approximately 10 times lower than the frequencies of antiviral B cells obtained by our unbiased screening approach (0.03–0.9%) 

Although both virus- and host-immune factors influence the levels of SARS-CoV-2 Ab production, the differences observed here appear to be largely dependent on pre-existing host-specific factors that distinguish HP from SP. Indeed, all subjects were recruited from the same hospital unit, over a short period, and HP infection was most likely acquired by providing routine care to SP. These criteria of selection reduce the risk of bias between the two groups due to virus-specific factors, such as the intervention of different viral strains. Whether these host-specific factors interfere on the magnitude of the humoral immune response against SARS-CoV-2 is unknown, but patients with severe COVID-19 are older and more frequently have comorbidities. Both advanced age and comorbidities are characterized by low-grade chronic systemic inflammation [12,13,26,27,28,29]. These patients have a systemic reduction in T cell functions, and hospitalized patients with underlying conditions have higher lymphopenia than those without [30,31,32,33]. To a lower extent, qualitative B cell defects have also been reported in SP [3,6,34]. Interestingly, a profound oligoclonal expansion of B cells with circulating oligoclonal bands has also been reported in SP [14,35,36]. Low-grade chronic systemic inflammation and resulting chronic immune defect with the SARS-CoV-2 induced lymphopenia that takes place in SP might influence the persistence of the viral antigens, and subsequently the magnitude of the anti-SARS-CoV-2 Ab response. 

Expanded B cell populations observed in SP seem to originate from extrafollicular B cell activation as observed in patients with systemic inflammatory/autoimmune diseases [36,37]. Extrafollicular B cell activation might also explain the higher levels of antiviral Ab, with altered T cell/B cell interactions. The optimal Ab highly specific response implies T and B cell interactions that are tightly regulated through key molecules, such as the CD40/CD40 ligand pathway, crucial for affinity maturation. Immune dysfunctions resulting from chronic inflammation affects this pathway and acute inflammation resulting from SARS-CoV-2 infection might further increase this defect. Severe COVID-19 is also characterized by an altered profile of cytokines that regulate Ab production. Levels of IL-10, critical for the differentiation of B cells into plasma cells secreting IgG and IgA, are dramatically elevated in SP and correlates with disease severity [38,39]. Whether Ab produced by extrafollicular activated B cells can confer protection against the virus or contribute to immunopathology by the development of polyreactive Ab or autoAb remains to be defined.

Another unexpected observation of our study was that low plasma titers of antiviral Ab are not predictive of the protective capacities conferred by circulating SARS-CoV-2 specific B cells. The determination of the neutralizing properties of the antiviral B cell repertoire of convalescent individuals showed that out of 204 supernatants, four B cell clones from the HP group were able to inhibit SARS-CoV-2 infection of BGM cells. These results were unexpected in view of the low levels of antiviral Ab detected in the plasma of these HP. They are in line with the importance of the quality/specificity/high affinity of the B cell response rather than the crude quantity [40]. 

The identification of circulating B cell clones with neutralizing properties in HP with low anti-RBD Ab levels suggests that they will be protected against reinfection with the same viral species. Consequently, they call for caution regarding the predictive estimations of the protective immunity conferred by natural infection and vaccines based only on the levels of circulating antiviral Ab.

In summary, our results highlight a qualitative defect of the regulation of the Ab synthesis in patients with severe forms of COVID-19. Importantly, they demonstrate also that low plasma titers of antiviral Ab are not necessarily synonym of low protection conferred by circulating B cells.

## 4. Materials and Methods

### 4.1. Study Participants

Ten convalescent volunteers were recruited at the Hospices Civils of Lyon (Lyon, France), from 30 April to 18 May 2020. Each patient had laboratory confirmed SARS-CoV-2 infection (positive RT-PCR test). Blood samples were collected in acid-citrate dextrose anticoagulant tubes, at least 2 weeks after the beginning of the symptoms. Eligible participants were health care personnel (*n* = 5) who recovered from a mild form of COVID-19 that did not necessitate hospitalization, and hospitalized patients (*n* = 5) who recovered from a severe form of the disease. All participants provided written informed consent before their participation to the study, and the study was approved by the institutional review board for ethics (Ethics Committee of the Hospitals of Lyon for the protection of people; IDRCB 2020-A01038-31). The study is registered on ClinicalTrials.gov (NCT04354766).

### 4.2. Generation of Immortalized B Cell Clones

Peripheral blood mononuclear cells (PBMCs) were isolated from human peripheral blood by density gradient centrifugation using Uni-sep maxi tubes (Eurobio Scientific, Les Ulis, France) at 400× *g* for 20 min, and washed twice with PBS. Stable circulating B cell clones were generated with an optimized technology based on CD40/CD40 ligand stimulation and EBV transformation (Human Blood B Booster kit DDX-HUBBB-Eurobio Scientific/Dendritics, Les Ulis, France). According to the manufacturer’s instructions, the day before the purification of PBMCs, 96 Ubottom microplates were seeded with 1 × 104 lyophilized FcgRII/CDw32 cells (cell line L4, Human Blood B Booster kit, Eurobio Scientific/Dendritics, Les Ulis, France), and incubated overnight at 37 °C, in presence of 5 µg/mL of anti-CD40 Ab (Mab89, Eurobio Scientific/Dendritics, Les Ulis, France). Purified PBMCs were resuspended in booster reagent medium (Eurobio Scientific/Dendritics, Les Ulis, France) and 1 × 104 cells/well were cocultured with FcgRII/CDw32 cells during 8 days at 37 °C, in presence of 1µg/mL of monoclonal B cell stimulating Ab (clones 107B5 and 110F7, Eurobio Scientific/Dendritics, Les Ulis, France) and EBV suspension (Human Blood B Booster kit, Eurobio Scientific/Dendritics, Les Ulis, France). At day 3, cellular patches were enumerated in each well, in order to define the number of initial B cell clones. At day 8, plates were duplicated and supplemented with 1 × 104 cells/well of lyophilized fibroblastic L6 cells stably expressing the CD40 ligand (Human Blood B Booster kit, Eurobio Scientific/Dendritics, Les Ulis, France), 1µg/mL of 107B5 and 110F7 Ab, and EBV suspension. At day 21 of culture, supernatants were screened for the presence of anti-SARS-CoV-2 Ab. Positive cultures were cloned until a monoclonal stage was obtained.

### 4.3. Indirect Immunofluorescence Assay

B cell clone supernatants were screened by indirect immunofluorescence staining on SARS-CoV-2–infected BGM cells fixed with ice-cold acetone (95%). After incubation with supernatants (30 min at 37 °C), cells were washed with PBS and further incubated with FITC- anti-human IgG, A, M Ab (Abliance, Compiègne, France) at 1:300 dilution. Plasma titers of anti-SARS-CoV-2 Ab were determined by serial dilutions. The titer was defined as the reciprocal of the highest positive dilution. Cells were imaged, using an Axioplan 2 imaging microscope (Zeiss, Munich, Germany).

### 4.4. Rapid Antibody Test

The Wantai SARS-CoV-2 Ab Rapid Test (Wantai Biological Pharmacy, Beijing, China) was carried out with the plasma of the convalescent patients in accordance with the manufacturer’s instructions. Although this test is not a quantitative test, we performed a semi-quantitative measurement from 0 to 4+, according to the strength of the line, with 4+ corresponding to an intensity equivalent to the control line [22].

### 4.5. ELISA

The ELISAs for the detection of plasma anti-RBD (Wantai SARS-CoV-2 Total Ab, Wantai Biological Pharmacy, Beijing, China) and anti-NP specific Ab (SARS-CoV-2 NP Ab ELISA, ImmunoDiagnostics, Sha Tin, Hong-Kong) were performed according to the manufacturer’s instructions. Antigenic specificities of the Ab produced by the immortalized B cell clones were determined with “in house” ELISAs. Ninety-six–well ELISA plates were coated overnight at room temperature with 1 µg/mL of the following recombinant SARS-CoV-2 proteins (Clinisciences, Nanterre, France) diluted in carbonate buffer: RBD, S1, S2, NP, envelope, helicase, and papain-like protease protein. After three washes with PBS–0.05% Tween 20 (PBST), 100 μl of diluted supernatants (1:3) in PBST–1% BSA were added and incubated for 2 h at 37 °C. After washing three times with PBST, plates were incubated with 200-fold diluted peroxidase-conjugated goat anti-human Ig G, A, M (Abliance, Compiègne, France) for 2 h at 37 °C. Plates were revealed by adding 100 μL of TMB (Eurobio Scientific/Dendritics, Les Ulis, France) after three washing steps in PBST. After 30 min incubation, ODs were measured at 620 nm, using an automated microplate reader (Sunrise, Tecan, Switzerland). The characterization of the isotypes, the subclasses, and the light chains of the Ab produced by immortalized B cell clones were performed by ELISA, using specific Ab and HRP-labeled anti-human Ig Ab (Thermo Fisher Scientific, Dardilly, France).

### 4.6. Seroneutralization Assay

A plaque reduction neutralization test was used for the detection of neutralizing Ab. Supernatants (tested in duplicate) were mixed at equal volume with the live SARS-CoV-2 virus (RoBo strain, Saint-Etienne, France) [41]. After gentle shaking for 30 min at room temperature, 150 µL of the mix was transferred into 96-well microplates covered with Vero-E6 cells. The plates were incubated at 37 °C in a 5% CO_2_ atmosphere. Measurements were obtained microscopically 5–6 days later when the cytopathic effect of the virus control reached 100 TCID50/150 µL. Neutralization was recorded if more than 50% of the cell layer was preserved. 

### 4.7. Statistical Analysis

All statistical analyses were carried out using Prism version 8 software (GraphPad software, San Diego, CA, U.S.A.). Differences between groups were analyzed using Mann–Whitney test for continuous variables. Categorical variables were compared using the two-sided Fisher’s exact test. A *p*-value less than 0.05 was considered statistically significant.

## Figures and Tables

**Table 1:** Antiviral B cell responses of convalescent SARS-CoV-2 infected patients.

	Health Care Personnel ^a^	Severe Patients ^a^	*p*-Value
N	5	5	
Sex (M/F)	2/3	4/1	0.524
Age, years; median (min–max)	54 (27–60)	81 (48–91)	0.048
> 65 years (%)	0 (0)	4 (80)	0.048
Hypertension (%)	1 (20)	5 (100)	0.048
Diabetes mellitus (%)	0 (0)	2 (40)	0.444
Obesity (%)	1 (20)	3 (60)	0.524
Symptoms onset to sampling, days; median (min–max)	39 (27–45)	20 (15–38)	0.032
Antiviral Ab titers ^b^, log_10_ (IIF); median (min–max)	1.7 (1.4–2.2)	2.3 (2.2–2.6)	0.024
Anti-RBD total Ab, (rapid test) ^c^; median (min–max)	0 (0–1)	3 (0–4)	0.048
Anti-RBD total Ab titers, log_10_ (ELISA); median (min–max)	0.9 (0.8–0.9)	1.9 (1.7–2.2)	0.008
Anti-NP total Ab titers, log_10_ (ELISA); median (min–max)	1.7 (0.1–2.4)	2.8 (2.6–3.6)	0.008
Frequency of antiviral B cell clones ^d^; median (min–max)	0.5 (0.3–1.8)	1.0 (0.3–9.0)	0.309

^a^ Infection by SARS-CoV-2 was confirmed by RT-PCR. ^b^ Plasma titers were determined by indirect immunofluorescence staining on SARS-CoV-2 infected BGM cells. ^c^ Semi-quantitative measurement of plasma anti-RBD antibodies were performed using the Wantai SARS-CoV-2 Ab rapid Test. According to the strength of the line, the signal was graded from 0 to 4+ [22]. ^d^ Frequencies of specific B cells against SARS-CoV-2 among 277,500 B cell clones screened by IIF on infected cells. For each sample, the frequency of specific B cells clones corresponds to the ratio of positive clones against SARS-CoV-2 versus the total number of clones generated by immortalization. The frequencies are expressed as the number of positive B cell clones per 1000 B cells. Abbreviations: IIF—indirect immunofluorescence; NP—nucleocapsid protein; RBD—receptor binding domain of the spike protein.

**Table 2:** Protective B cell clones isolated from convalescent patients.

Patient	Screened B Cell Clones	SARS-CoV-2 Reactive B Cell Clones	Neutralizing B Cell Clones	Clone	Mono-Clonal	Oligo-Clonal	Isotype	Antigenic Specificity
HP2	45,000	26	3	3E7	✔		IgG3κ	RBD^+^S1^+^ S2^−^NP^−^E^−^PP^−^
				2G5.B1		✔	IgAM,κ	RBD^−^S1^+^ S2^−^NP^−^E^−^PP^−^
				2D11.E1	✔		IgAκ	RBD^+^S1^−^ S2^−^NP^−^E^−^PP^−^
HP4	15,500	28	1	5B9.A1		✔	IgGAMκ	RBD^−^S1^−^ S2^−^NP^−^E^−^PP^−^
SP2	25,000	38	3	3D9		✔	IgMκ	RBD^−^S1^−^ S2^−^NP^−^E^−^PP^−^
				3G10.A2		✔	IgG4κ, IgMλ	RBD^−^S1^−^ S2^−^NP^−^E^−^PP^−^
				1F4.C2	✔		IgG4κ	RBD^+^S1^+^ S2^−^NP^−^E^−^PP^−^
SP4	49,000	15	3	3F7.F1	✔		IgG4κ	RBD^+^S1^+^ S2^−^NP^−^E^−^PP^−^
				3H2.F1	✔		IgG1κ	RBD^+^S1^+^ S2^−^NP^−^E^−^PP^−^
				2H2.F10	✔		IgG1κ	RBD^+^S1^+^ S2^−^NP^−^E^−^PP
SP5	36,000	34	1	3B12.A6		✔	IgGAMκ	RBD^+^S1^+^ S2^−^NP^−^E^−^PP

Antibodies reactivity was evaluated against the following viral antigens: RBD, S1, S2, nucleoprotein, envelope, helicase, and papain-like protease protein. Abbreviations: E—envelope, H—helicase, HP—health care personnel, NP—nucleoprotein, PP—papain-like protease protein, SP—severe patients.

## Data Availability

The datasets generated for this study are available on request to the corresponding author.

## Supplementary Materials

The following are available online at https://www.mdpi.com/article/10.3390/ijms222011095/s1, Table S1: Individual demographical data and antiviral B cell responses of convalescent SARS-CoV-2 infected patients, Figure S1: Correlation between the frequency of circulating SARS-Cov-2 B cells and the plasma levels of antiviral antibodies.
