# Supplementary material for: Impact of Host Immune Status on Discordant Anti-SARS-CoV-2 Circulating B Cell Frequencies and Antibody Levels
Source: Int J Mol Sci. 2021 Oct 14;22(20):11095. doi: 10.3390/ijms222011095 (PMC8540683; doi:10.3390/ijms222011095)
Supplement: Supplementary file 1 [file ijms-22-11095-s001.zip › ijms-1403811-supplementary.pdf]

SUPPLEMENTAL TABLE

	Sex (M/F)	Age (y)	Age (>65 ans)	Hyper-tension	Diabetes mellitus	Obesity	Symptom onset to sampling (days)	Antiviral Ab titers, log10 (IIF)[a]	Anti-RBD total Ab (rapid test)[b]	Anti-RBD total Ab titers, log10 (ELISA)	Anti-NP total Ab titers, log10 (ELISA)	Frequency of antiviral B cell clones[c]
**SP1**	M	90	Yes	Yes	No	No	20	2,3	4	1,7	3,6	9,00
**SP2**	M	91	Yes	Yes	No	Yes	17	2,2	0	1,7	2,8	1,56
**SP3**	F	81	Yes	Yes	No	Yes	15	2,6	2	1,9	2,7	1,05
**SP4**	M	72	Yes	Yes	Yes	Yes	26	2,6	4	2,1	3,5	0,31
**SP5**	M	48	No	Yes	Yes	No	38	2,2	3	2,2	2,6	0,94
**HP1**	F	60	No	No	No	No	27	2,2	1	0,9	1,9	0,45
**HP2**	M	27	No	No	No	No	30	1,7	0	0,9	2,4	0,58
**HP3**	M	27	No	No	No	No	39	1,4	0	0,8	0,1	0,30
**HP4**	F	60	No	Yes	No	Yes	41	1,7	0	0,9	1,7	1,81
**HP5**	F	54	No	No	No	No	45	1,4	0	0,9	0,1	0,36

Supplementary Table S1: Individual demographical data and antiviral B cell responses of convalescent SARS-CoV-2 infected patients

[a] Plasma titers were determined by indirect immunofluorescence staining on SARS-CoV-2 infected BGM cells. [b] Semi-quantitative measurement of plasma anti-RBD antibodies were performed using the Wantai SARS-CoV-2 Ab rapid Test. According to the strength of the line, the signal was graded from 0 to 4+. [c] Frequencies of specific B cells against SARS-CoV-2 among 277,500 B cell clones screened by IIF on infected cells. For each sample, the frequency of specific B cells clones corresponds to the ratio of positive clones against SARS-CoV-2 versus the total number of clones generated by immortalization. The frequencies are expressed as the number of positive B cell clones per 1000 B cells. Abbreviations: IIF: indirect immunofluorescence; NP: nucleocapsid protein; RBD: receptor binding domain of the spike protein.

SUPPLEMENTAL FIGURE

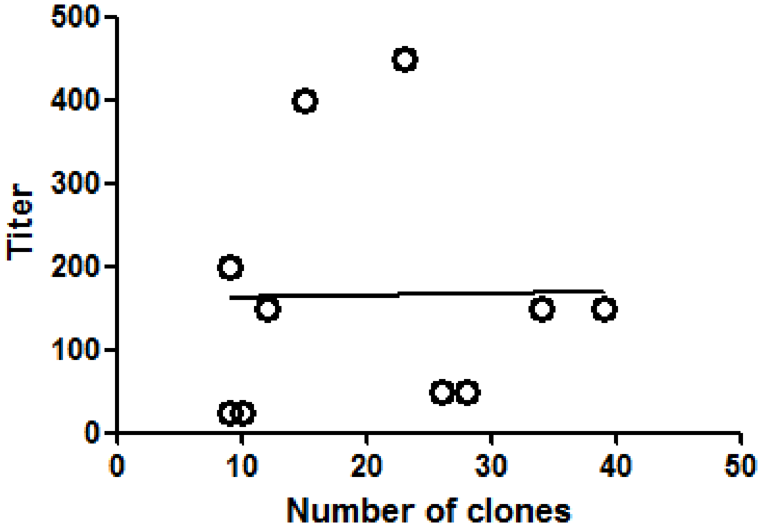

Supplementary Figure S1: Correlation between the frequency of circulating SARS-Cov-2 B cells and the plasma levels of antiviral antibodies. Correlations were calculated between the frequencies of specific B cell clones and antiviral antibodies titers of each plasma, determined by indirect immunofluorescence on SARS-CoV-2 infected cells.
